# Exercise and Sports Metabolomics: Analytical Platforms, Metabolite Annotation, Pathway-Level Interpretation, and Biomarker-Panel Readiness

**DOI:** 10.3390/metabo16080530

**Published:** 2026-07-27

**Authors:** Donghai Lin, Yifen Chen, Caihua Huang

**Affiliations:** 1 Key Laboratory for Chemical Biology of Fujian Province, High-Field NMR Research Center, College of Chemistry and Chemical Engineering, Xiamen University, Xiamen 361005, China; cyf1303280@163.com; 2 Research and Communication Center of Exercise and Health, Xiamen University of Technology, Xiamen 361024, China; huangcaihua@xmut.edu.cn

**Keywords:** exercise metabolomics, sports metabolomics, metabolite identification, metabolite annotation, quality control, pathway validation, biomarker-panel readiness

## Abstract

Exercise and sports metabolomics provide a systems-level approach to characterizing how acute exercise, training adaptation, nutrition, recovery, and environmental stress reshape human metabolism. By profiling metabolites related to substrate utilization, mitochondrial function, redox balance, inflammation, muscle stress, and recovery kinetics, these approaches can reveal pathway-level responses that conventional single biomarkers cannot capture. However, many exercise-responsive features remain difficult to interpret because of incomplete chemical identification, uncertain annotation confidence, limited quantitative reproducibility, variable pre-analytical control, inconsistent data processing, and insufficient biological validation. This narrative review examines recent advances in exercise and sports metabolomics, with emphasis on LC–MS, GC–MS, NMR spectroscopy, IMS–MS, and CE–MS workflows; platform selection; metabolite annotation and identification; pathway-level interpretation; and evidence requirements for candidate-panel development. Exercise-responsive metabolites should be interpreted as context-dependent pathway signals rather than isolated indicators of fatigue, recovery, adaptation, or performance. The review consolidates requirements for sampling, quality control, metadata capture, repeated-measures analysis, and external validation within an evidence-readiness roadmap. Wearable biochemical monitoring, AI-assisted analysis, and multi-omics integration may support future applications, but their value depends on analytical robustness, external validation, and physiological interpretability. Exercise and sports metabolomics should therefore progress from descriptive feature discovery toward reproducible, quantitatively reliable, and biologically validated pathway-level interpretation.

## 1. Introduction

Exercise elicits coordinated metabolomic and lipidomic responses that are better interpreted as molecular patterns than as isolated single-marker changes [1,2,3,4,5]. These responses vary with sample matrix, analytical platform, sport discipline, nutritional status, sex, training phase, and recovery window [1,5,6,7,8,9,10,11,12]. Metabolomics, the comprehensive analysis of small molecules in biological systems, can characterize the biochemical effects of acute exercise, training adaptation, nutrition, recovery, and environmental stress [1,2,3,4,5]. Metabolite profiles link phenotype to substrate availability, energy demand, mitochondrial function, redox balance, endocrine strain, inflammation, tissue remodeling, guthost interactions, and environmental exposure [1,2,3,4,5,13,14,15,16,17,18,19,20,21,22,23,24,25,26,27,28,29,30,31].

The field now faces a methodological and interpretive bottleneck. Many studies identify statistically altered metabolite features after exercise. However, fewer establish transparent annotation, chemical identification when required, quantitative reliability, or biological meaning. Fewer still demonstrate consistency across sample matrices, analytical platforms, sport disciplines, nutritional states, sex, training phases, and recovery windows [5,32,33,34,35,36,37,38]. The key question is therefore not simply whether exercise alters the metabolome, but which metabolite patterns can be reliably measured, interpreted within defined biological contexts, and developed into useful candidate panels.

Unlike traditional single-biomarker approaches, metabolomics can capture coordinated changes across multiple metabolic pathways. Complementary platforms, including liquid chromatography–mass spectrometry (LC–MS), gas chromatography–mass spectrometry (GC–MS), nuclear magnetic resonance (NMR) spectroscopy, ion mobility spectrometry–mass spectrometry (IMS–MS), and capillary electrophoresis–mass spectrometry (CE–MS), can profile glycolytic intermediates, tricarboxylic acid (TCA)-cycle metabolites, amino acids, acylcarnitines, ketone bodies, lipids, volatile organic compounds, and related stress signals [13,14,15,16,17,18,19,20,21,22,23,24,25,26,27,28,29,30,31,39,40,41,42,43,44,45,46]. This analytical breadth creates opportunities for pathway-level interpretation, but it also increases the risk of overinterpreting individual markers. Signals such as lactate, β-hydroxybutyrate (βHB), acetylcarnitine, creatine kinase, branched-chain amino acids (BCAAs), and inflammatory lipid mediators do not have uniform meanings across exercise settings. Their interpretation depends on exercise modality, intensity, duration, training status, carbohydrate availability, sex, circadian timing, recent training load, environmental exposure, sample matrix, and recovery interval [13,14,15,16,17,18,19,20,21,22,23,24,25,26,27,28,29,30,31]. Exercise and sports metabolomics are therefore most informative when these signals are interpreted as context-dependent pathway responses rather than isolated indicators of fatigue, recovery, adaptation, or performance.

In this review, exercise metabolomics and sports metabolomics are treated as related but distinct contexts. Exercise metabolomics primarily concerns acute exercise, exercise interventions, training adaptation, nutrition, recovery, and environmental stress, with emphasis on general physiological mechanisms. Sports metabolomics focuses more specifically on athletes and sport-specific settings, including training load, competition demands, performance-related function, recovery monitoring, and applied interpretation. The combined term “exercise and sports metabolomics” is used only for analytical, interpretive, and validation issues that apply to both contexts.

Previous reviews provide important foundations but differ in emphasis from the present synthesis. Systematic reviews have addressed the scope of exercise and sports metabolomics, standardization challenges, non-invasive sampling, metabolite annotation, and multi-omics integration [5]. Platform-focused and methodological reviews have considered LC–MS, GC–MS, NMR spectroscopy, IMS–MS, and CE–MS workflows [39,40,41,42,43,44,45,46]. Wearable-sensing reviews have primarily examined near-real-time biochemical monitoring rather than broad metabolomics [47,48,49,50,51,52,53,54,55,56]. Multi-omics studies and reviews have emphasized integration across metabolomics, lipidomics, proteomics, transcriptomics, epigenomics, microbiome profiling, and physiological data [57,58,59]. Building on this literature, the present review focuses on analytical confidence, quantitative robustness, biological validation, and evidence readiness.

Accordingly, this review synthesizes advances in exercise and sports metabolomics since 2020 through a pathway-centered and evidence-readiness framework. We distinguish three evidence levels: athlete-centered human studies with direct relevance to exercise adaptation, recovery, and applied interpretation; controlled exercise studies in healthy non-athlete populations that clarify general physiological responses; and indirect mechanistic evidence from disease, aging, preclinical, or technology-development contexts. This hierarchy is intended to prevent overextension of exploratory or indirect findings while still recognizing their value for mechanistic interpretation and hypothesis generation.

The review addresses four questions. First, which analytical platforms are most appropriate for general exercise-physiology questions, sport-specific applications, and different sample matrices? Second, how do annotation, chemical identification, quantification, quality control, and data processing affect biological inference? Third, which metabolite patterns have plausible pathway-level meaning across exercise modalities, nutritional states, and recovery phases? Fourth, what level of validation is required before candidate metabolites can be organized into reproducible panels? Wearable biochemical sensing, AI-assisted analysis, and multi-omics integration are considered as supporting tools whose utility depends on analytical robustness, external validation, and physiological interpretability.

The review makes three contributions. First, it prioritizes pathway-level interpretation and staged evidence readiness over isolated exercise-responsive metabolite claims. Second, it distinguishes athlete-centered evidence, controlled healthy-exercise evidence, and indirect mechanistic evidence. Third, it integrates platform selection, analytical confidence, pathway interpretation, wearable monitoring, AI-assisted analysis, multi-omics context, and staged validation into a single framework. Across the reviewed literature, the clearest conclusion is that exercise metabolomics is most informative when coordinated pathway responses are interpreted alongside exercise context. Athlete-centered and controlled exercise studies most consistently support changes in carbohydrate flux, lipid and ketone metabolism, amino acid turnover, endocrine and inflammatory signaling, and muscle-stress pathways. Direct translation into individualized performance, fatigue, or recovery decisions nevertheless remains immature [1,2,3,4,5,7,12,20].

### Methodological Approach and Evidence-Readiness Framework

This narrative review was informed by a structured literature-search and evidence-synthesis strategy. PubMed, Scopus, and Web of Science were searched for English-language, peer-reviewed articles published between January 2020 and March 2026. Search terms combined concepts related to exercise or sports metabolomics with recovery, fatigue, overreaching, training adaptation, substrate utilization, analytical platforms, metabolite identification and annotation, wearable biochemical sensing, artificial intelligence, and multi-omics integration. Reference lists of key reviews and highly relevant original studies were also screened to identify additional literature. Recent original human studies, methodological investigations, consensus or reporting guidance, and reviews directly relevant to the analytical and interpretive scope of the manuscript were prioritized. Earlier foundational publications were retained where necessary to support established analytical standards, quality-control procedures, metabolite-annotation frameworks, or athlete-monitoring principles.

Because the purpose of this narrative review was to support critical and practical interpretation rather than to conduct a formal systematic review or meta-analysis, evidence was weighted according to its relevance to exercise and sport applications. Level 1 evidence comprised athlete-centered human studies directly relevant to training load, recovery, substrate utilization, fatigue, adaptation, or performance monitoring. Level 2 evidence comprised controlled human exercise studies in healthy non-athlete populations; these studies help clarify general physiological responses but require caution when extrapolated to trained or elite athletes. Level 3 evidence comprised disease, aging, preclinical, technology-development, and other indirect mechanistic studies. Level 3 studies were used for pathway interpretation and hypothesis generation but were not treated as direct evidence for routine athlete monitoring or management.

This evidence-readiness framework was used to reduce subjective overinterpretation and distinguish mechanistically informative findings from application-ready biomarkers. A metabolite or metabolite panel was considered most relevant to applied sport when it demonstrated biological plausibility, acceptable analytical reproducibility, longitudinal stability, and an association with sport-relevant outcomes such as recovery kinetics, training adaptation, fatigue status, substrate availability, or performance-related function.

## 2. Analytical Foundations for Exercise and Sports Metabolomics

Both exercise metabolomics and sports metabolomics should begin with a clearly defined biological or applied question rather than maximal feature detection alone. The analytical value of LC–MS, GC–MS, NMR spectroscopy, IMS–MS, and CE–MS depends on coverage, sensitivity, and resolution. It also depends on whether the workflow supports transparent annotation, chemical identification where required, reproducible quantification, quality control, and pathway-level interpretation. Because exercise-induced metabolic profiles are strongly context-dependent, relevant protocol, participant, and athlete metadata should be recorded according to the study question and interpreted within the evidence-readiness framework developed in later sections.

This review considers LC–MS, GC–MS, NMR spectroscopy, IMS–MS, and CE–MS as complementary analytical approaches. LC–MS, GC–MS, and NMR spectroscopy are established metabolomics platforms. IMS–MS is an emerging complementary separation dimension coupled to MS, whereas CE–MS expands coverage of highly polar and charged metabolites. Platform selection should match the exercise question, sample matrix, target metabolite class, quantification strategy, and validation stage. LC–MS is well suited to broad discovery, lipidomics, and targeted validation [39,40]. GC–MS is useful for breath volatile organic compounds, volatile or derivatized metabolites, organic acids, and fatty acids [41,42]. NMR spectroscopy supports reproducible repeated-measures quantification of abundant metabolites [43]. IMS–MS improves discrimination among isobaric, co-eluting, and structurally related compounds [44]. CE–MS provides complementary separation of polar and charged metabolites, including amino acids, organic acids, nucleotides, and phosphorylated intermediates [45,46]. Table 1 summarizes these complementary roles.

Recent analytical advances have expanded the detection, quantification, annotation, and identification of exercise-responsive metabolites. However, analytical performance alone does not establish biological relevance. An exercise-responsive feature becomes interpretable only when its annotation confidence is transparently reported, its identity is confirmed when required by the claim, and its measurement is reproducible across relevant batches or matrices. Pathway-level interpretation also requires control of biological and pre-analytical variables that can alter metabolite direction and magnitude. For example, elevated ketone bodies may reflect prolonged exercise, fasting, carbohydrate restriction, or fat adaptation. Altered amino acid profiles may reflect substrate use, dietary intake, muscle repair, or systemic stress. Platform-specific findings should therefore be interpreted in relation to matrix choice, sampling time, annotation confidence, identification confidence, and validation stage [39,40,41,42,43,44,45,46].

In this review, metabolite annotation and metabolite identification are distinct terms. Annotation refers to assigning a putative metabolite name or class with a stated confidence level. Supporting evidence may include accurate mass, isotope pattern, MS/MS similarity, spectral-library matching, retention behavior, collision cross-section values, or pathway plausibility. Identification is reserved for higher-confidence confirmation of chemical identity, ideally supported by authentic standards, retention-time matching, diagnostic MS/MS spectra, internal standards where appropriate, or orthogonal targeted assays. Untargeted features should therefore not be described as identified metabolites unless this higher evidentiary threshold has been met.

**Figure 1:**
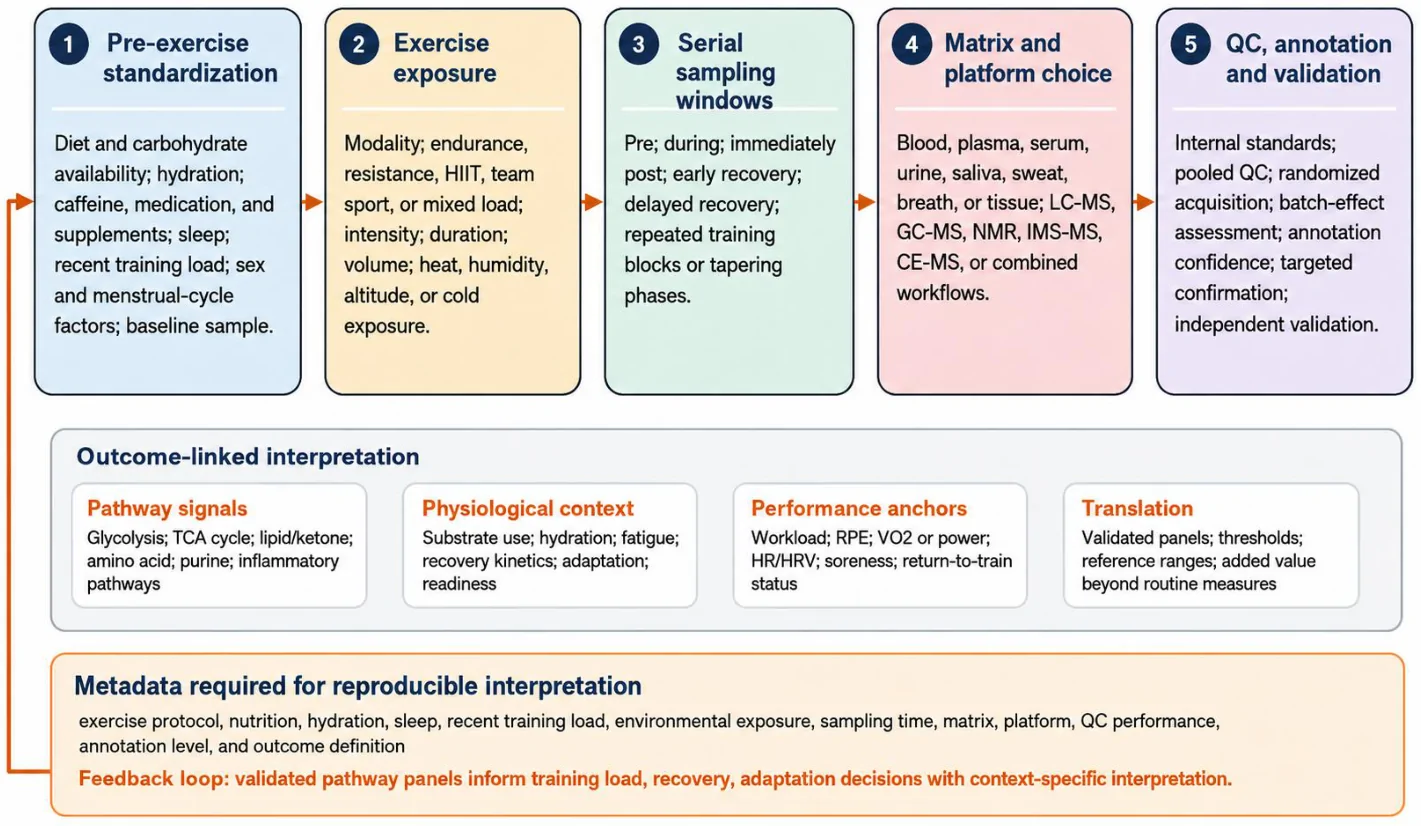
Exercise- and sport-specific metabolomics workflow. The workflow begins with pre-exercise standardization, definition of exposure, and serial sampling across acute and recovery windows. Biological matrices and analytical platforms are selected according to the target metabolite classes, study population, and validation stage. Quality-control procedures and annotation confidence support pathway-level interpretation. Physiological and performance outcomes then determine whether metabolite signals are relevant to substrate use, fatigue, recovery, adaptation, and athlete readiness, and whether candidate panels warrant further validation.

Figure 1 places these analytical choices within an exercise-specific metabolomics workflow. It links pre-exercise standardization, exercise-exposure definition, serial sampling, matrix and platform selection, quality control, metabolite annotation, pathway-level interpretation, and physiological or performance outcomes. Together, these elements provide the methodological basis for the evidence-readiness and validation framework developed in later sections.

### 2.1. LC–MS–Based Metabolomics

LC–MS has become a central platform in exercise and sports metabolomics because it combines high analytical sensitivity with broad coverage of chemically diverse metabolites [39,40]. It can capture amino acids, organic acids, acylcarnitines, ketone bodies, bile acids, lipid mediators, and complex lipids. These metabolite classes can inform fuel selection, mitochondrial substrate handling, metabolic flexibility, inflammatory responses, and recovery-related remodeling. LC–MS is most informative when exploratory profiling is linked to a defined biological question and followed by transparent annotation, chemical identification where required, quantification, and validation.

LC–MS is particularly well suited to broad discovery, lipidomics, substrate-use profiling, recovery-kinetics analysis, and targeted validation of pathway-linked candidates. Its value is greatest when serial blood, urine, sweat, saliva, or tissue samples are aligned with predefined workloads, nutritional conditions, recovery windows, and functional outcomes.

Recent advances in sample preparation, chromatographic separation, mass accuracy, spectral acquisition, and metabolite annotation have expanded LC–MS capabilities. However, standardized pre-analytical handling remains essential. Variation in blood collection, urine normalization, storage, extraction, and batch processing can be mistaken for exercise-induced metabolic change when not controlled or reported [60,61]. Miniaturized and microfluidic extraction methods may reduce sample requirements and processing time, which is useful for repeated sampling before, during, and after exercise [62]. High-resolution Orbitrap and time-of-flight instruments have improved feature detection, isotope-pattern recognition, adduct discrimination, and MS/MS-based structural characterization [63,64]. Greater feature detection, however, does not necessarily strengthen biological interpretation. Without pooled quality-control samples, internal standards, randomized acquisition order, batch-effect assessment, and transparent precision reporting, large feature tables may contain uncertain or poorly reproducible findings.

A major development in LC–MS-based exercise metabolomics is the progression from untargeted discovery toward reproducible quantification. Untargeted workflows can reveal unexpected exercise-responsive features and pathways. Targeted and semi-targeted assays are better suited to confirming chemical identity and quantifying candidate metabolites across repeated samples, training phases, matrices, and validation cohorts. LC–MS studies should report annotation levels, MS/MS evidence, retention-time information where available, missingness, matrix effects, normalization procedures, analytical precision, and quality-control performance. Putatively annotated features based on accurate mass, predicted spectra, or pathway plausibility should be clearly distinguished from confidently identified metabolites supported by authentic standards or equivalent confirmatory evidence [65]. This distinction is particularly important when metabolites are used to support claims about mitochondrial function, substrate oxidation, inflammation, or recovery biology.

Spectral libraries, in silico fragmentation tools, and lipid-annotation workflows have strengthened the interpretation of LC–MS data, particularly for lipidomics, acylcarnitines, bile acids, and related metabolite classes [66,67]. Advances in large-scale lipid annotation and metabolite-structure prediction have also improved coverage and candidate prioritization [68,69]. Nevertheless, computational annotation should support hypothesis generation rather than substitute for chemical identification. When a candidate metabolite underpins pathway interpretation or candidate-panel development, confirmation should include appropriate MS/MS spectra, retention-time matching, authentic or internal standards, targeted assays, and independent replication.

LC–MS has been used to characterize exercise-related changes in lipid metabolism, amino acid turnover, acylcarnitines, TCA-cycle intermediates, lactate- and pyruvate-related metabolites, and inflammatory lipid mediators across high-intensity interval and endurance exercise [1,6,7]. These signals can inform glycolytic stress, fatty acid oxidation, mitochondrial substrate handling, amino acid utilization, and inflammatory remodeling. Their interpretation nevertheless remains context-dependent. For example, acylcarnitines may reflect mitochondrial substrate pressure, but their meaning differs during prolonged endurance exercise, low-carbohydrate availability, incomplete recovery, or high training load. Similarly, lipid mediators may indicate adaptive tissue remodeling or persistent inflammatory stress when elevations extend across recovery windows.

Future LC–MS studies should move beyond reporting differential features and instead develop reproducible, pathway-informed candidate panels. Such panels should integrate annotation confidence, identification status, quantitative precision, quality-control performance, sampling context, and physiological metadata. Relevant metadata include exercise modality, nutritional status, sampling time, recovery phase, and functional outcomes. Progress will depend less on detecting additional features than on generating confidently annotated or identified metabolites and biologically interpretable pathway signatures.

### 2.2. GC–MS-Based Metabolomics

GC–MS remains valuable in exercise and sports metabolomics because it provides robust chromatographic separation, mature spectral libraries, and reliable analysis of volatile, semi-volatile, and derivatized metabolites [41,42]. It is particularly useful for breath volatile organic compounds (VOCs), organic acids, fatty acids, and selected metabolites related to substrate use, oxidative stress, and environmental exposure. As a complement to LC–MS, GC–MS can expand coverage of metabolite classes that are otherwise difficult to characterize, especially in serial breath or urine samples collected across exercise and recovery.

GC–MS is most appropriate when the study objective is to profile breath VOCs, volatile or semi-volatile compounds, derivatized organic acids, fatty acids, or related metabolites. Acute exercise and recovery studies require standardized breath or urine collection, appropriate blanks, retention-index information, spectral-library matching, and authentic standards where feasible.

Advances in derivatization chemistry, solid-phase microextraction, chromatographic resolution, and breath VOC analysis have improved the sensitivity and reproducibility of GC–MS-based exercise metabolomics [70,71,72]. Breath acetone and isoprene may provide insight into lipid oxidation, ketone metabolism, and isoprenoid-related pathways during exercise. However, VOC concentrations are influenced by diet, ventilation, hydration, airway physiology, microbiome activity, ambient contamination, sampling devices, storage conditions, and data processing. These variables should be controlled or reported to prevent technical or environmental variation from being misinterpreted as exercise-induced metabolic change.

GC–MS can provide high-confidence annotation and, for selected compounds, chemical identification when retention indices, high-quality mass spectra, spectral-library matching, and authentic standards are available. This capability is particularly useful for derivatized polar metabolites, organic acids, fatty acids, and selected VOCs. Nevertheless, derivatization efficiency, sample stability, matrix effects, carryover, and batch variation remain important sources of analytical variability. GC–MS workflows should therefore include standardized collection protocols, blanks, pooled quality-control samples, internal or isotopically labeled standards where feasible, randomized acquisition order, and transparent reporting of annotation confidence, identification criteria, and data-processing procedures.

In exercise metabolomics, GC-MS can support mechanistic interpretation of substrate use, lipid oxidation, oxidative stress, and environmental exposure during controlled exercise and recovery protocols. In sports metabolomics, breath and urine GC-MS may provide time-sensitive information relevant to athlete monitoring, but these signals require calibration against sport-specific context, exercise intensity, respiratory exchange ratio, nutritional status, recovery kinetics, and established biochemical markers [73,74,75]. Future work should prioritize harmonized breath and urine sampling protocols, cross-platform validation, and longitudinal designs that determine whether GC-MS-derived metabolites can contribute to reproducible pathway interpretation and biomarker-panel development.

### 2.3. NMR-Based Metabolomics

NMR spectroscopy is valuable in exercise and sports metabolomics because it provides highly reproducible, quantitative, and non-destructive measurements [43]. It is also well suited to longitudinal repeated-measures designs. Compared with many MS-based methods, NMR generally has lower sensitivity and narrower metabolite coverage. Nevertheless, it can robustly quantify abundant metabolites, including lactate, glucose, amino acids, ketone bodies, creatine-related metabolites, urea, and organic acids. These characteristics make NMR particularly useful for tracking metabolic trajectories during acute exercise, training blocks, tapering, recovery, and environmental exposure.

NMR is most appropriate when quantitative comparability and longitudinal stability are more important than maximal feature coverage. It is well suited to repeated sampling during acute exercise, hydration shifts, recovery kinetics, training-block monitoring, tapering, and environmental-stress studies. Its main advantage is the reliable characterization of within-individual trajectories, which may be more informative than cross-sectional group differences.

Advances in spectral processing, automated quantification, spectral assignment, and multivariate analysis have improved the detection of reproducible metabolic changes in plasma, serum, urine, saliva, and sweat. Reliable interpretation nevertheless depends on careful control of sample handling, pH, dilution, urine normalization, water suppression, spectral alignment, baseline correction, and metabolite quantification. Studies should therefore report preprocessing parameters, normalization methods, excluded spectral regions, and annotation or identification confidence.

NMR has been applied to endurance exercise, resistance exercise, high-intensity interval exercise, hypoxia, heat exposure, hydration, substrate use, amino acid turnover, and recovery-related metabolic trajectories [8,9,10,43,76]. Changes in lactate, glucose, urea, creatinine, amino acids, ketone bodies, and dehydration-related profiles can inform interpretation of metabolic stress, fluid balance, substrate availability, and recovery biology. However, these signals are also influenced by diet, time of day, recent exercise, biofluid dilution, training phase, and environmental conditions. Standardized sample collection, spectral processing, normalization, and reporting of pre-analytical variables therefore remain essential [77,78].

Computational and machine-learning approaches may support pattern recognition, feature extraction, and classification of NMR-based metabolic trajectories [79]. However, they should not substitute for analytical reproducibility, transparent feature selection, biological plausibility, or external validation. Miniaturized and low-field NMR systems may improve accessibility, but they do not yet match standard laboratory platforms in sensitivity, resolution, or quantitative reliability [80]. Overall, NMR is best positioned as a complementary platform for reproducible quantification and longitudinal pathway interpretation when serial profiles are integrated with exercise context, hydration measures, dietary records, recovery outcomes, and functional testing.

### 2.4. IMS–MS as an Emerging Complementary MS Dimension

Compared with the established LC–MS, GC–MS, and NMR workflows, IMS–MS remains an emerging complementary dimension in exercise and sports metabolomics. When coupled to mass spectrometry, it provides gas-phase ion separation and collision cross-section information. These data improve discrimination among isobaric, co-eluting, and structurally related metabolites [2,44,81]. IMS–MS should therefore be regarded as a complementary tool for strengthening structural annotation and refining candidate metabolite panels, rather than as a routine standalone platform for athlete monitoring.

IMS–MS is most valuable when structural discrimination is the principal analytical bottleneck. Relevant applications include lipidomics, acylcarnitine profiling, steroid-like compounds, and other structurally related metabolites in plasma, urine, sweat, and tissue extracts. In these settings, ion-mobility separation can resolve species that remain difficult to distinguish by mass-to-charge ratio, retention time, and fragmentation spectra alone. This added discrimination may improve interpretation of substrate metabolism, oxidative stress, inflammation, and post-exercise recovery.

Recent high-resolution IMS-MS workflows can reduce chemical noise and improve selectivity [82]. Collision cross-section values can complement accurate mass, retention time, and MS/MS spectra when assigning metabolite identities. However, IMS-MS-derived evidence should support transparent annotation and, where required, chemical identification rather than substitute for them. This distinction is critical because premature assignment of lipids, acylcarnitines, or steroid-like metabolites can lead to incorrect pathway-level interpretation.

IMS–MS remains less standardized than LC–MS, GC–MS, and NMR spectroscopy. Wider application will require expanded collision cross-section libraries, harmonized data-processing workflows, cross-platform comparison, and transparent reporting of annotation confidence and identification evidence. Instrument cost, database limitations, data-analysis complexity, and limited interlaboratory standardization also constrain its current adoption. Its contribution should therefore be judged by whether the added structural information improves pathway interpretation, targeted validation, and reproducible candidate-panel development.

### 2.5. CE–MS for Polar and Charged Metabolite Profiling

CE–MS provides high-efficiency separation of highly polar and charged metabolites from relatively small sample volumes [45,46]. It is well suited to amino acids, organic acids, nucleotides, phosphorylated metabolites, and other energy-related intermediates that may be incompletely resolved by other analytical workflows. CE–MS can therefore complement LC–MS and GC–MS in studies of exercise-associated energy metabolism, purine turnover, amino acid utilization, and recovery-related metabolic change. However, migration-time reproducibility, interface stability, sensitivity, lower lipid coverage, limited interlaboratory standardization, and comparatively limited adoption currently constrain its routine use. CE–MS is thus best positioned as a complementary platform for polar and ionic metabolite profiling, particularly for targeted or semi-targeted validation.

CE–MS is most useful when studies require targeted or semi-targeted profiling of polar and ionic metabolites from serial plasma, urine, sweat, or tissue samples. It may be particularly informative for questions involving energy metabolism, nucleotide and purine turnover, amino acid utilization, and recovery-related pathway changes. Reliable implementation requires standardized sample preparation, migration-time control, quality-control assessment, appropriate internal standards, and matrix-specific analytical validation. CE–MS-derived signals should be interpreted alongside the exercise protocol, sampling time, nutritional context, and relevant physiological or functional outcomes. Until reproducibility and cross-laboratory comparability improve, CE–MS should remain a complementary tool for pathway interpretation and validation rather than a routine standalone platform for athlete monitoring [45,46].

## 3. Biological Interpretation, Evidence Readiness, and Pathway-Level Validation

A major limitation of many exercise metabolomics studies, and of sports metabolomics studies that extrapolate from them, is insufficient biological interpretation rather than insufficient analytical sensitivity. Lists of altered metabolites rarely establish whether observed changes reflect acute energy demand, substrate availability, adaptive remodeling, incomplete recovery, inflammatory stress, nutritional status, or analytical variation [1,2,3,4,5,13,14,15,16,17,18,19,20,21,22,23,24,25,26,27,28,29,30,31]. Exercise-responsive metabolites should therefore be interpreted as pathway-embedded, context-dependent signals rather than isolated biomarkers. Their meaning depends on the exercise exposure, sampling matrix, sampling time, nutritional state, training background, and analytical confidence.

**Figure 2:**
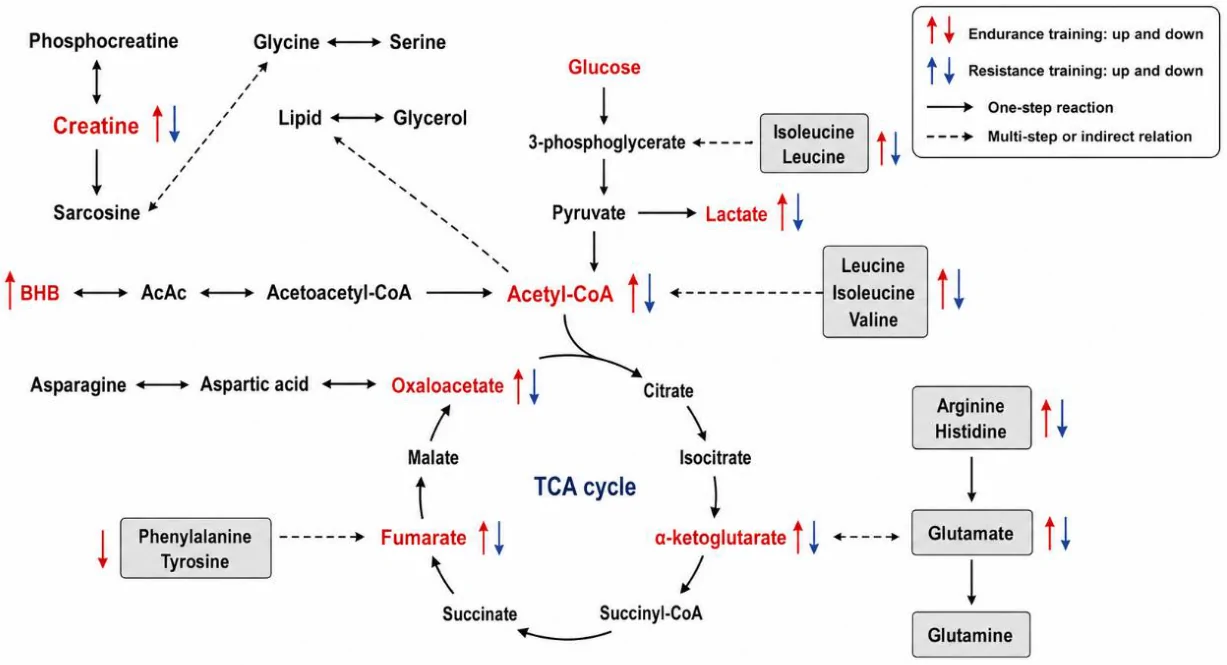
Pathway-level context for representative exercise-responsive metabolites. The diagram depicts selected interactions among glycolysis, the TCA cycle, lipid and ketone body metabolism, amino acid metabolism, and related energy- and stress-associated pathways. Upward and downward arrows represent a conceptual synthesis of commonly reported post-exercise tendencies. Glycolytic and carbohydrate-related responses are supported by [13,14,15,16]; ketone-body responses by [17,18]; mitochondrial substrate handling, acylcarnitines, and TCA-cycle metabolism by [19,20,21]; amino acid and BCAA metabolism by [20,22]; and endocrine, inflammatory, neuroimmune, and muscle-stress context by [23,24,25,26,27,28,29,30,31]. These arrows are not quantitative pooled estimates or universal response directions. Their direction and magnitude depend on exercise modality, intensity, duration, training status, nutritional state, sample matrix, sampling time, and recovery phase. The figure therefore serves as a framework for pathway-level interpretation rather than a comprehensive metabolic map or a fixed biomarker scheme.

Figure 2 provides a pathway-level framework for interpreting interactions among glycolysis, the TCA cycle, lipid and ketone body metabolism, amino acid metabolism, and related energy- and stress-associated pathways. The metabolite classes shown are supported by representative literature on glycolytic regulation and carbohydrate availability [13,14,15,16], ketone-related substrate use [17,18], mitochondrial substrate handling [19,20,21], amino acid and BCAA metabolism [20,22], endocrine and inflammatory responses [23,24,25], tryptophan–kynurenine metabolism [26,27,28,29], and muscle-stress markers [30,31]. High-intensity exercise may produce rapid changes in glycolytic and purine-related metabolites, whereas prolonged endurance exercise may induce broader shifts in lipid oxidation, amino acid turnover, oxidative stress, and inflammatory mediators [13,14,15,16,17,18,19,20,21,22,23,24,25]. The direction and magnitude of these responses vary with exercise modality, intensity, duration, nutritional state, training status, sample matrix, and sampling time [13,14,15,16,17,18,19,20,21,22,23,24,25,26,27,28,29,30,31].

Figure 2 should therefore be read as a conceptual framework rather than a universal response map. Lactate may reflect glycolytic flux, altered clearance, or incomplete recovery; βHB may reflect prolonged exercise, fasting, carbohydrate restriction, or fat adaptation; and BCAA may reflect amino acid oxidation, dietary intake, muscle repair, or systemic stress [13,14,15,16,17,18,19,20,21,22]. These examples illustrate why pathway-level interpretation should precede any candidate-biomarker claim.

### 3.1. Pathway-Centered Interpretation and Candidate Biomarker Readiness

A pathway-centered framework is essential for distinguishing mechanistic interpretation from biomarker overstatement. Table 2 presents representative exercise-responsive metabolites alongside complementary physiological markers. Lactate, glucose, pyruvate, βHB, amino acids, acylcarnitines, and TCA-cycle intermediates are metabolites measurable with appropriate analytical workflows. In contrast, cortisol, IL-6, creatine kinase, and myoglobin are complementary endocrine, inflammatory, and muscle-stress markers rather than metabolites in the analytical metabolomics sense. The table also distinguishes signals commonly measurable in routine workflows from mechanistic intermediates that are difficult to quantify directly. Lactate, pyruvate, and glucose inform glycolytic flux, carbohydrate availability, and redox-linked energy metabolism [13,14,15,16]. βHB informs ketone metabolism and substrate availability [17,18]. Acylcarnitines, acetylcarnitine, citrate, and related TCA-cycle intermediates provide information on mitochondrial substrate handling, fatty acid oxidation, anaplerosis, and metabolic remodeling [19,20,21]. Amino acids and BCAAs reflect substrate use, nitrogen metabolism, muscle repair, and nutrition [20,22]. Cortisol, IL-6, tryptophan-kynurenine metabolites, creatine kinase, and myoglobin provide complementary endocrine, inflammatory, neuroimmune, and muscle-stress context [23,24,25,26,27,28,29,30,31].

Across representative exercise studies, lactate, glucose, and related glycolytic metabolites are among the most consistently measured indicators of acute carbohydrate flux when workload and sampling time are standardized [13,14,15,16]. Prolonged endurance exercise also produces coordinated changes in βHB, acylcarnitines, TCA-cycle intermediates, and amino acids, consistent with substrate switching, mitochondrial substrate handling, and amino acid turnover [17,18,19]. Glycolytic regulation, mitochondrial energy metabolism, lipid and ketone metabolism, and amino acid metabolism therefore represent recurrent pathway-level responses across exercise modalities. However, the direction and magnitude of individual responses remain context dependent and should not be treated as universal biomarker thresholds.

The most reproducible value of exercise metabolomics currently lies in pathway-level synthesis. Table 2 therefore separates directly measured metabolites from complementary physiological markers and mechanistic intermediates that are difficult to quantify in routine biofluid workflows. Lactate, pyruvate, glucose, βHB, amino acids, acylcarnitines, citrate, and selected TCA-cycle intermediates can often be quantified by targeted workflows when pre-analytical handling is appropriate [13,14,15,16,17,18,19,20,21,22]. Acetyl-CoA is biologically central but is mainly intracellular and difficult to measure directly in most circulating biofluids. Its interpretation is usually inferred from related metabolites, including citrate, ketone bodies, acylcarnitines, and other TCA-cycle intermediates [19,20,21]. Labile intermediates, particularly oxaloacetate, require careful sample handling, matrix-specific validation, and targeted confirmation before supporting pathway-level claims [46,47,48]. Cortisol, IL-6, creatine kinase, and myoglobin should be interpreted as physiological context markers rather than metabolomic biomarkers [23,24,25,26,27,28,29,30,31].

No single metabolite can reliably define fatigue, overreaching, recovery readiness, adaptation, or performance potential. Candidate markers should instead be assessed for biological plausibility, analytical reproducibility, and longitudinal relevance. Biological plausibility requires involvement in a pathway meaningfully engaged by the exercise stimulus. Analytical reproducibility requires reliable measurement across batches, instruments, matrices, and sampling conditions. Longitudinal relevance requires that a signal tracks within-individual changes across training, recovery, competition, or intervention periods more consistently than random day-to-day variation. These criteria are essential because exercise-responsive metabolites are influenced by diet, sleep, circadian timing, infection, psychological stress, environmental exposure, sex, recent training load, sample matrix, and sampling time.

Exercise modality further shapes pathway interpretation. Endurance exercise often produces coordinated changes in carbohydrate oxidation, fatty acid oxidation, TCA-cycle activity, ketone metabolism, amino acid turnover, and oxidative stress [1,7,20]. Resistance exercise and high-intensity interval exercise may more strongly perturb glycolytic metabolism, lactate-pyruvate dynamics, purine metabolism, amino acid turnover, and muscle-stress markers [1,7,20]. Repeated resistance training may also induce mitochondrial, lipid-related, and amino acid-associated remodeling, whereas prolonged endurance exercise may involve inflammatory signaling, muscle stress, and delayed recovery kinetics. These patterns should not be interpreted as fixed biomarker rules. Reliable interpretation requires integration of exercise modality, intensity, duration, training background, nutritional status, sample matrix, sampling time, and recovery phase.

Fatigue, overreaching, and recovery are multi-system states rather than single-metabolite abnormalities. Tryptophan-kynurenine metabolism may inform neuroimmune stress and fatigue-related pathways [26,27,28,29], whereas IL-6 and cortisol provide inflammatory and endocrine context [23,24,25]. Amino acid profiles may reflect substrate use, nutrition, or muscle repair [20,22]. Lactate clearance, acylcarnitines, creatine kinase, and myoglobin may collectively inform energy stress, mitochondrial substrate pressure, muscle strain, or incomplete recovery [15,20,21,30,31]. Their value lies in supporting pathway-level interpretation within a defined exercise and biological context, rather than in guiding standalone prescriptive decisions.

Temporal kinetics are also central to recovery metabolomics. A metabolite measured immediately after exercise may reflect acute energetic demand, whereas the same metabolite measured 3, 24, or 48 h later may reflect clearance, repair, persistent stress, substrate redistribution, or maladaptation. Lactate and glucose may inform acute substrate flux and carbohydrate availability [13,14,15,16], whereas βHB, acylcarnitines, amino acids, and TCA-cycle intermediates may help characterize substrate switching, mitochondrial substrate handling, energy restoration, and post-exercise remodeling [11,12,17,18,19,20,21,22,83,84,85,86]. Serial sampling across defined recovery windows should therefore be linked to functional outcomes, including power restoration, soreness, neuromuscular performance, recovery scores, and subsequent training quality.

Interpretation of substrate use and oxidative metabolism also requires caution. Pyruvate and lactate are associated with glycolytic flux and anaerobic demand [13,15,16], whereas βHB more often reflects ketone metabolism, carbohydrate availability, and fat-adapted substrate use [17,18]. Acetylcarnitine and other acylcarnitines provide information on fatty acid oxidation and mitochondrial substrate pressure [21,85,86]. Citrate, acetyl-CoA, and related TCA-cycle intermediates inform oxidative metabolism, anaplerosis, and mitochondrial remodeling [12,19,83,84,86]. None of these signals directly equates to performance or adaptation. Their interpretation should be integrated with respiratory exchange, power output, heart rate, perceived exertion, dietary intake, training load, recovery context, and analytical confidence.

Candidate metabolite panels should be interpreted as context-dependent pathway signatures rather than universal thresholds. Discovery-stage features are hypothesis-generating; validation-ready candidates require appropriate identification, quantitative reproducibility, and independent replication; and application-ready panels require longitudinal reliability, external validation, predefined physiological or functional outcomes, and evidence of added value.

### 3.2. Mechanistic Context and Evidence Boundaries

Exercise metabolomics should prioritize analytically robust and biologically interpretable evidence from human exercise studies. Sports metabolomics additionally requires validation in trained or competitive populations and sport-relevant settings. Research in aging biology, metabolic disease, immunometabolism, tissue regeneration, and preclinical exercise models can provide valuable mechanistic insight into mitochondrial biogenesis, lipid handling, amino acid metabolism, inflammatory resolution, redox regulation, guthost metabolism, and inter-organ crosstalk [87]. However, findings from these adjacent contexts should remain hypothesis-generating until they are validated in physically active or athlete populations using standardized exercise protocols, defined sampling windows, and relevant physiological or functional outcomes.

For conceptual clarity, this review assigns evidence to three levels according to study population, exercise specificity, sampling design, analytical confidence, and linkage to sport-relevant outcomes. Level 1 evidence comprises athlete-centered human studies in which trained or competitive participants undertake sport-relevant exercise exposures, sampling windows are defined, and metabolic changes are linked to training load, recovery, substrate use, fatigue, adaptation, or performance-related outcomes. Level 2 evidence comprises controlled human exercise studies in healthy non-athlete populations. These studies support general physiological inference but require validation before extrapolation to trained or elite athletes. Level 3 evidence comprises disease, aging, preclinical, tissue-specific, technology-development, and other indirect mechanistic studies. Such studies support pathway plausibility and hypothesis generation but should not be used as direct evidence for athlete monitoring or performance decision-making [57,58,59,87,88,89]. Table 3 summarizes the assignment criteria, appropriate inferences, limitations, and validation requirements for each evidence level. Importantly, these levels constitute an evidence-directness and sport-applicability framework; they do not represent a formal assessment of methodological quality, risk of bias, or certainty of evidence. Rather, they indicate how directly a study can inform sport-specific interpretation and application.

A key limitation across all levels is that circulating metabolites are not simple proxies for tissue-specific biology. Plasma, serum, urine, saliva, sweat, and breath reflect integrated contributions from skeletal muscle, liver, adipose tissue, immune cells, gut-derived metabolism, renal handling, pulmonary exchange, and the circulating compartment itself. For example, a circulating acylcarnitine or amino acid signal may reflect muscle substrate pressure, hepatic metabolism, adipose lipolysis, immune activation, guthost metabolism, clearance kinetics, or matrix-specific handling rather than a single tissue source. Multi-omics studies of human skeletal-muscle adaptation further show that exercise responses are sex-, modality-, tissue-, and pathway-specific [57]. Circulating metabolites should therefore be interpreted in relation to tissue biology, training background, sample matrix, and exercise context. Integrative metabolomics, lipidomics, proteomics, transcriptomics, epigenomics, microbiome profiling, and computational modeling may help identify relevant pathway networks [57,58,59]. At present, however, these approaches are most useful for mechanism discovery, pathway prioritization, and candidate-panel hypothesis generation rather than direct training prescription or individualized decision-making.

Clear evidence boundaries are particularly important for exercise-mimetic and disease-derived metabolic research. Metabolites, pathways, or molecular signatures identified outside athlete-centered or controlled human exercise studies should be described as mechanistic candidates until replicated in relevant exercise settings. Such evidence may inform hypotheses about mitochondrial remodeling, inflammatory control, substrate flexibility, tissue repair, guthost metabolism, or metabolic resilience. It should not, however, be presented as direct support for performance optimization, recovery management, field-based monitoring, or sport-specific intervention strategies [88,89]. Future studies should determine whether these candidate pathways remain reproducible, biologically interpretable, and functionally relevant under standardized exercise conditions. Accordingly, this review uses adjacent evidence to strengthen pathway-level interpretation and guide future validation, not to justify immediate applied recommendations.

### 3.3. Wearable Biochemical Monitoring and AI-Assisted Metabolomics: Distinct Roles and Validation Requirements

Wearable biochemical monitoring and AI-assisted metabolomics serve different functions within the exercise-monitoring workflow and should not be treated as equivalent technologies. Most wearable systems do not provide broad metabolomic coverage. Instead, they measure a limited number of analytes, such as sweat lactate, glucose, electrolytes, pH, cortisol, or selected breath volatile organic compounds, in field-compatible or near-real-time settings [15,47,48,49,50,51,52,53,54,55]. They are therefore better described as biochemical monitoring technologies that can increase sampling density and provide physiological context, rather than as metabolomics platforms equivalent to LC–MS, GC–MS, NMR spectroscopy, IMS–MS, or CE–MS. Their interpretation is affected by sweat rate, skin contamination, sensor drift, temperature, hydration status, sampling site, breathing pattern, device calibration, matrix effects, and lag relative to blood, interstitial fluid, or muscle metabolism.

Validation of wearable biochemical monitoring should be analyte- and matrix-specific. Continuous glucose monitoring (CGM), for example, measures interstitial rather than blood glucose. Its interpretation in non-diabetic athletes depends on sensor accuracy during movement, physiological lag, calibration stability, exercise intensity, nutritional context, and linkage to defined physiological outcomes [14,56]. Wearable lactate monitoring is also promising but should not be assumed to reproduce blood lactate kinetics, because local sweat-gland metabolism, sweat rate, sampling location, skin contamination, and sensor architecture can affect the measured signal [15]. Before physiological interpretation, wearable assays should be compared with appropriate reference laboratory methods. Validation should include calibration procedures, analytical precision, limits of detection where relevant, sensor drift, matrix effects, repeated-measures reproducibility, and field robustness. Agreement should also be assessed across exercise intensities, hydration states, temperatures, and recovery windows.

AI-assisted metabolomics is a computational support layer rather than a measurement platform. It may assist feature prioritization, integration of metabolomic, physiological, and training-load data, response-phenotype classification, metabolite annotation, and hypothesis generation for targeted validation [90,91,92,93]. However, AI cannot substitute for robust experimental design, analytical validation, or biological interpretation. Exercise and sports metabolomics datasets are often small, heterogeneous, and strongly context dependent, increasing the risk of overfitting, training-test leakage, unstable feature selection, and poor generalizability. Model reporting should therefore include sample size, cohort characteristics, preprocessing and normalization procedures, feature-selection strategy, missing-data handling, confounder control, internal and external validation, calibration performance, uncertainty assessment, and biological plausibility checks [94,95].

The near-term value of wearable biochemical monitoring and AI-assisted metabolomics lies in context enrichment, feature prioritization, and validation support rather than autonomous decision-making. Wearable outputs should be linked to reference assays, repeated-measures reproducibility, and physiological or functional outcomes, including power output, perceived exertion, heart rate, heart-rate variability, sleep, dietary intake, recovery scores, and subsequent training quality [96,97,98,99]. AI-derived candidate panels and prediction models require independent external validation across relevant sports, sex groups, training phases, nutritional states, environmental conditions, biological matrices, and analytical platforms. Both approaches should therefore be positioned as validation-dependent tools that improve data density and interpretation, rather than as standalone metabolomic biomarkers or independent decision systems.

## 4. Barriers to Validation and Future Research Priorities

The central translational challenge is to move from descriptive metabolite discovery toward evidence-ready pathway interpretation and candidate-panel development. The priorities outlined below address study design, analytical validation, longitudinal modeling, sport-specific interpretation, and outcome-linked testing [5,20,34,35,36,37,38].

Before data acquisition, future studies should define the biological question, target population, sample matrix, sampling time course, and validation outcome. These design choices determine whether a study is primarily exploratory, analytically validated, longitudinally informative, or implementation-ready [5,37,38].

This framework also clarifies the current translational boundary of the field. The available literature supports metabolomics as a valuable tool for mechanistic interpretation and hypothesis generation. However, application-ready biomarker panels require repeated-measures validation, cross-platform confirmation, context-specific reference ranges, and demonstrated links to physiological or functional outcomes before routine individualized use can be justified.

### 4.1. Large-Scale, Sport-Specific, and Longitudinal Studies

Study designs should move beyond isolated pre-post comparisons toward dense repeated-measures frameworks. Recommended approaches include individualized baseline profiling during stable training, standardized sampling before and after defined workloads, serial sampling across recovery, and follow-up through intensified loading, tapering, competition, travel, environmental exposure, and return-to-training phases [20,34,35,36,37]. Sampling windows should match the expected biology of the question. Acute substrate flux may require sampling over minutes to hours, recovery and muscle-stress responses over 24–72 h, and training adaptation over weeks to months.

Longitudinal studies should combine metabolomics with standardized covariate control and appropriate statistical modeling. Diet, carbohydrate availability, hydration, caffeine, medication or supplement use, sleep, menstrual-cycle phase where relevant, and recent training load should be controlled or recorded. External-load measures, including distance, speed, power, volume, and session structure, should be considered alongside internal-load measures, including heart rate, perceived exertion, heart-rate variability, soreness, recovery scores, and relevant biochemical or physiological reference measures. Mixed-effects models, within-subject baselines, repeated-measures reliability estimates, and predefined physiological or performance outcomes can help distinguish metabolic adaptation from day-to-day variation, batch effects, and uncontrolled contextual influences.

Sport-specific interpretation is essential because an identical metabolite pattern may have different biological meanings across endurance, sprint, resistance, team, combat, and mixed-modality sports. Large-scale cohorts may not always be feasible in elite athletes because of limited populations, travel, competition schedules, and restricted access. In these settings, dense longitudinal sampling, rigorous within-subject baselines, standardized workloads, and clearly defined functional outcomes may yield more informative evidence than simply increasing sample size. Future studies should therefore balance cohort size with sampling density, protocol control, ecological validity, and outcome linkage.

### 4.2. Key Research Gaps and Validation Priorities

Several research gaps continue to limit biological interpretation, cross-study comparability, and validation in exercise and sports metabolomics. Table 4 prioritizes these gaps according to evidence maturity and summarizes the supporting literature. Nutritional context and long-term training adaptation are two particularly important priorities for applied exercise and sport settings. They should be addressed as staged validation targets rather than as general methodological recommendations.

Dietary strategies are a major source of metabolic variation in exercise and sports metabolomics [101,102,103]. Carbohydrate periodization, ketogenic or low-carbohydrate diets, intermittent fasting, supplementation, and low energy availability can substantially reshape metabolite profiles. Without adequate control or documentation, these factors may be misinterpreted as training adaptation, incomplete recovery, or altered metabolic flexibility. Future studies should therefore combine metabolomics with validated dietary records, standardized pre-sampling nutrition, and, where feasible, objective indicators of substrate use, glycogen availability, ketone metabolism, and energy balance.

Long-term training adaptation remains less well characterized than acute post-exercise metabolic responses [5,11,106]. Chronic adaptations, including mitochondrial remodeling, altered lipid utilization, substrate flexibility, inflammatory resolution, and sustained exercise-induced metabolic plasticity, require longitudinal sampling across training cycles. Future studies should link metabolite trajectories to training load, recovery kinetics, dietary context, environmental exposure, health status, performance testing, and athlete-reported outcomes. Such designs are needed to distinguish durable training adaptation from transient responses to a single exercise bout.

Environmental and logistical stressors also require greater attention. Heat, altitude, humidity, travel-related circadian disruption, sleep loss, and competition stress can alter substrate metabolism, hydration status, oxidative stress, inflammatory signaling, and recovery kinetics. Although these exposures are highly relevant to real-world training and competition, they remain underrepresented in controlled metabolomics studies. Ecologically valid studies should determine which metabolite patterns reflect adaptive acclimatization and which are associated with excessive physiological strain, impaired recovery, or increased vulnerability to illness or injury [104,105].

Methodological harmonization is equally important. Variation in sample collection, biofluid handling, metabolite extraction, analytical workflows, normalization, batch-effect correction, metabolite annotation and identification, metadata capture, and reporting standards to limit reproducibility across studies [38]. Exercise-responsive metabolomic signals are also sensitive to sampling time, fasting status, recent exercise, hydration, menstrual-cycle phase, medication or supplement use, and environmental exposure. These variables should be systematically documented and, where appropriate, incorporated into statistical analyses. Harmonized workflows will improve confidence that reported metabolite differences reflect biological variation rather than pre-analytical or technical artifacts.

### 4.3. Interdisciplinary Priorities for the Next Phase of the Field

The next phase of exercise and sports metabolomics will require closer collaboration among analytical chemists, exercise physiologists, sports nutritionists, data scientists, clinicians, coaches, and athletes. Metabolomic signals become biologically meaningful only when interpreted in relation to the exercise protocol, training history, nutritional status, recovery state, environmental exposure, functional testing, and, in sports metabolomics, athlete-specific context [107,108,109,110,111]. Integrated metabolomics and lipidomics are particularly useful for linking biochemical signatures to substrate selection, mitochondrial remodeling, lipid utilization, inflammatory responses, and exercise adaptation [2].

Interdisciplinary studies should distinguish discovery, replication, longitudinal validation, and implementation testing. This evidence hierarchy reduces overstatement, improves reproducibility, and enables readers to determine whether a finding is exploratory, mechanistically informative, validation-ready, or sufficiently mature for applied testing.

Multi-omics integration offers an important opportunity but should be guided by biological questions rather than data accumulation alone. Integrating metabolomics with lipidomics, proteomics, transcriptomics, epigenomics, microbiome profiling, wearable physiological data, biomechanics, and nutrition records may help identify pathway-level and inter-organ mechanisms underlying exercise adaptation. Large-scale multi-omics studies also demonstrate the potential to identify sex-, tissue-, and modality-specific molecular responses to exercise [57]. However, integrated models require biological plausibility, interpretability, and external validation before they can support individualized interpretation or candidate-panel development.

Future studies should also determine whether metabolomics provides information beyond established physiological and contextual measures. Candidate metabolite panels should be evaluated against training load, heart-rate variability, perceived exertion, sleep, dietary intake, environmental exposure, recovery kinetics, performance testing, and health-related outcomes. This outcome-linked approach can establish whether metabolomics improves mechanistic explanation, recovery assessment, nutritional interpretation, or detection of excessive physiological strain beyond simpler monitoring tools. The field will be most useful when metabolomic data are analytically robust, biologically interpretable, longitudinally validated, and integrated with practical exercise and health-related context.

## 5. Translating Exercise and Sports Metabolomics: An Evidence-Readiness Roadmap Section

Progress from discovery profiling to evidence-ready application should follow staged validation rather than direct translation of exploratory findings. Table 5 links each validation stage to its purpose, required analytical, biological, and statistical evidence, expected output, and readiness level. The framework comprises four progressive stages: discovery; analytical validation and independent replication; longitudinal validation; and implementation testing. It distinguishes hypothesis-generating features from validation-ready candidates and application-ready biomarker panels. This translational-maturity roadmap is complementary to the evidence-directness and sport-applicability framework in Table 3. Whereas Table 3 indicates how directly a study informs sport-specific interpretation, Table 5 indicates the maturity of a finding for translation to applied use.

Table 5 provides the detailed criteria for movement between these stages and for distinguishing hypothesis-generating features, validation-ready candidates, and application-ready panels. This approach also aligns with athlete-monitoring frameworks, which emphasize that training adaptation, fatigue, and recovery should be interpreted using integrated longitudinal information rather than isolated measurements [96,112,113].

Implementation-oriented studies should define the biological or applied question before sample collection. Recovery kinetics, substrate utilization, heat stress, altitude adaptation, low energy availability, overreaching, and nutritional interventions require different sampling windows, matrices, covariates, and outcome measures. Repeated-measures designs with individualized baselines, standardized workloads, and serial recovery sampling should be used where feasible, while diet, recent training load, sleep, hydration, sex, menstrual-cycle phase where relevant, environmental exposure, and sampling time should be controlled or recorded. Studies of low energy availability should interpret metabolite signals alongside training load, nutritional intake, endocrine status, and clinical risk frameworks; studies of recovery or overreaching should include functional, perceptual, and biochemical outcomes [114,115,116].

For applied interpretation, candidate panels should be tested against predefined functional and physiological outcomes and compared with established measures, including training load, heart-rate variability, perceived exertion, sleep, dietary intake, and performance testing. Independent external validation should assess reproducibility across relevant sex groups, sports, competitive levels, nutritional and environmental contexts, matrices, and analytical platforms; computational models should also demonstrate calibration and generalizability. Context-specific decision thresholds should be considered only after analytical reliability, biological plausibility, longitudinal validity, and practical feasibility are established.

This roadmap positions metabolomics as a mechanistic and validation-oriented layer within integrated exercise and sports science rather than an isolated decision tool. Its near-term contribution is explanatory, clarifying pathway responses to training, nutrition, recovery, environmental stress, and competition. Table 5 provides the criteria needed before a candidate panel can support applied use.

## 6. Conclusions

Exercise metabolomics has advanced substantially since 2020 through improvements in LC–MS, GC–MS, NMR spectroscopy, IMS–MS, CE–MS, wearable biochemical monitoring, AI-assisted analysis, and multi-omics integration. Sports metabolomics builds on this foundation but requires additional validation in athlete-centered, sport-specific settings. The central challenge is not feature detection alone, but determining which signals are transparently annotated, appropriately identified, quantitatively reproducible, mechanistically interpretable, longitudinally reliable, and biologically validated.

At present, most exercise-responsive metabolite signatures remain exploratory or mechanistically informative rather than ready for routine individualized interpretation or intervention planning. Lactate, glucose, acylcarnitines, βHB, amino acids, TCA-cycle intermediates, and complementary markers such as creatine kinase, myoglobin, cortisol, IL-6, and tryptophan-kynurenine-related metabolites can support pathway-level interpretation. Their meaning, however, depends on exercise modality, intensity, sampling time, nutritional status, sex, training phase, environmental exposure, biological matrix, analytical platform, and individual baseline variability.

Future progress should follow the staged evidence-readiness roadmap in Table 5. Among the more reproducibly measured exercise responses, lactate and glucose provide useful indicators of acute carbohydrate flux when workload and sampling time are standardized [13,14,15,16]. Across prolonged endurance exercise, coordinated changes in ketone bodies, acylcarnitines, TCA-cycle intermediates, and amino acids recurrently support interpretation of substrate switching, mitochondrial substrate handling, and amino acid turnover [17,18,19,20,21,22]. These response patterns should be interpreted as context-dependent pathway signals rather than universal biomarker thresholds. Detailed readiness requirements are consolidated in Table 5; until these criteria are met, such signatures should remain candidate rather than application-ready biomarker panels.

Wearable biochemical monitoring, AI-assisted modeling, and multi-omics integration may support this progression when they are calibrated, externally validated, biologically plausible, and transparently reported. Used appropriately, exercise metabolomics can improve understanding of adaptation, recovery, substrate utilization, environmental stress, and maladaptive responses. Sports metabolomics can extend these insights toward athlete-centered interpretation only when candidate signatures are validated in sport-specific populations, sampling contexts, and performance-relevant outcomes.

## Figures and Tables

**Table 1:** Major metabolomics platforms used in exercise and sports science.

Platform	Suitable Matrices	Major Metabolite Coverage	Analytical Strengths	Key Limitations	Exercise-Specific Use and Validation Guidance
**LC–MS**	Blood, plasma, serum, urine, sweat, saliva, tissue extracts	Amino acids, organic acids, acylcarnitines, ketone bodies, bile acids, lipid mediators, complex lipids	High sensitivity; broad polar and non-polar coverage; compatible with targeted and untargeted workflows	Matrix effects; ion suppression; complex annotation; batch effects; need for standards and spectral libraries	Best suited for broad discovery, lipidomics, and targeted or semi-targeted quantification in plasma, serum, urine, sweat, saliva, or tissue extracts. Useful for acute exercise responses, recovery kinetics, substrate-use profiling, inflammatory lipid mediators, and targeted validation when supported by internal standards, pooled QC, MS/MS spectra, retention time, and authentic standards.
**GC–MS**	Blood, urine, breath, tissue extracts	Volatile/semi-volatile compounds, organic acids, fatty acids, derivatized polar metabolites	Mature spectral libraries; good chromatographic reproducibility; strong for VOCs and derivatized metabolites	Derivatization required for many polar metabolites; limited for thermolabile or large molecules; sample preparation can be labor-intensive	Best suited for breath VOCs, volatile or semi-volatile compounds, urine organic acids, fatty acids, and derivatized polar metabolites. Useful for substrate oxidation, lipid oxidation, oxidative stress, environmental exposure, and recovery-window studies when retention indices, spectral libraries, blanks, QC samples, and standards are used.
**NMR spectroscopy**	Plasma, serum, urine, saliva, sweat	Abundant metabolites, including lactate, glucose, amino acids, ketone bodies, creatine-related metabolites, urea, organic acids	Highly reproducible; quantitative; non-destructive; minimal preparation; suitable for repeated-measures studies	Lower sensitivity; limited coverage of low-abundance metabolites; spectral overlap	Best suited for longitudinal or repeated-measures studies requiring reproducible quantification of abundant metabolites in plasma, serum, urine, saliva, or sweat. Useful for hydration status, acute exercise response, recovery kinetics, training-block monitoring, and validation of stable quantitative markers, but less suitable for low-abundance discovery.
**IMS–MS**	Plasma, serum, urine, sweat, tissue extracts	Lipids, acylcarnitines, steroid-like compounds, isobaric or structurally similar metabolites	Adds collision cross-section information; improves separation of isobaric and co-eluting compounds	Limited standardization; database limitations; complex processing; high instrumentation cost	Best used as a complementary LC–MS or GC–MS dimension for lipidomics, acylcarnitines, steroid-like signals, and other isobaric, co-eluting, or structurally related metabolites. Useful for improving structural discrimination and refining biomarker panels, but not yet a routine standalone platform for exercise monitoring.
**CE–MS**	Plasma, serum, urine, sweat, and tissue extracts	Highly polar and charged metabolites, including amino acids, organic acids, nucleotides, and phosphorylated or energy-related intermediates	High-efficiency separation of ionic metabolites; low sample-volume requirements; complementary polar-metabolome coverage	Migration-time reproducibility; interface-dependent sensitivity; lower lipid coverage; limited interlaboratory standardization and adoption	Best suited for targeted or semi-targeted profiling of highly polar and charged metabolites in plasma, serum, urine, sweat, or tissue extracts, including amino acids, organic acids, nucleotides, and phosphorylated or other energy-related intermediates. Complementary for polar-metabolite validation where chromatographic workflows provide incomplete coverage.

**Note:** Table 1 summarizes major metabolomics platforms used in exercise and sports science, with platform-specific information supported by representative references for LC-MS [39,40], GC-MS [41,42], NMR spectroscopy [43], IMS-MS [44], and CE–MS [45,46]. Platform selection should be guided by the biological question, sample matrix, target metabolite classes, expected concentration range, sampling frequency, annotation or identification requirements, quantitative objectives, and validation strategy. LC-MS, GC-MS, NMR spectroscopy, IMS-MS, and CE-MS offer complementary strengths in coverage, sensitivity, quantitative reproducibility, structural discrimination, annotation and identification confidence, and polar-metabolite separation. No single platform is universally optimal. In practical exercise and sports metabolomics, Table 1 should be read as an operational guide. LC-MS is favored for broad discovery, lipidomics, and targeted validation; GC-MS for breath VOCs, volatile or derivatized metabolites, organic acids, and fatty acids; NMR spectroscopy for reproducible repeated-measures profiling; IMS-MS for complementary structural discrimination; and CE-MS for polar and charged metabolites.

**Table 2:** Representative exercise-responsive metabolites and complementary physiological markers for pathway-level interpretation and biomarker-readiness assessment.

Category/Marker Type	Representative Metabolite or Complementary Marker	Main Pathway or Physiological Context	Exercise-Related Interpretation	Analytical Status, Interpretation Cautions, and Supporting References
**Core metabolite: energy metabolism**	Lactate	Glycolysis; lactate-pyruvate redox balance; carbohydrate utilization	Commonly increases during high-intensity exercise and reflects increased glycolytic flux, but also depends on clearance, training status, carbohydrate availability, and sampling time.	Directly measurable in blood and selected wearable or biofluid workflows. Useful for interpreting glycolytic demand only when workload, sampling time, clearance kinetics, and recovery context are standardized [13,14,15,16].
**Core metabolite: energy availability**	Glucose	Glycolysis; glycogen availability; systemic energy homeostasis	Dynamic changes may reflect carbohydrate availability, hepatic glucose output, muscle uptake, nutrition, exercise duration, and recovery state.	Directly measurable in blood and interstitial-fluid workflows. Interpretation requires dietary records, exercise intensity, timing, and, where relevant, insulin or continuous-glucose-monitoring context [13,14].
**Core metabolite class: amino acid metabolism**	Branched-chain amino acids (BCAAs)	Amino acid metabolism; nitrogen balance; muscle protein turnover; substrate use	Changes may reflect amino acid oxidation, dietary intake, glycogen availability, muscle repair, or systemic stress. Direction and magnitude may differ by exercise modality and recovery phase.	Directly measurable by targeted metabolomics, but not specific for recovery or adaptation. Requires dietary control, repeated sampling, and interpretation within broader amino-acid and energy-metabolism panels [20,22].
**Complementary endocrine marker**	Cortisol	Endocrine stress response; glucose mobilization; protein and lipid metabolism	May increase with prolonged or high-intensity exercise, psychological stress, sleep disruption, energy deficit, or environmental strain.	Not a metabolite in the analytical metabolomics sense. Included as a complementary physiological marker; interpretation is limited by circadian rhythm, acute stress sensitivity, and sampling time [23].
**Metabolite class: neuroimmune metabolism**	Tryptophan/kynurenine-related metabolites	Tryptophan-kynurenine pathway; immune regulation; neuroimmune stress	May reflect prolonged exercise stress, inflammation, immune activation, or fatigue-related neuroimmune processes.	Directly measurable as metabolites, but pathway interpretation is influenced by inflammation, infection, stress, diet, and training load. Validation should include repeated sampling and functional outcomes [26,27,28,29].
**Complementary inflammatory marker**	IL-6	Exercise-induced cytokine signaling; immunometabolism; inflammatory regulation	Can increase after prolonged or intense exercise and may reflect both metabolic signaling and inflammatory stress.	Not a metabolite; included as a complementary inflammatory marker. Should be interpreted according to timing, exercise modality, muscle glycogen status, tissue stress, and recovery phase [24,25].
**Complementary muscle-stress marker**	Creatine kinase	Muscle membrane disruption; phosphocreatine-related energy metabolism; tissue stress	Circulating levels may increase after muscle-damaging exercise, especially eccentric, resistance, or repeated high-intensity work. Responses are highly variable among individuals.	Not a metabolite; included as a complementary muscle-stress marker. Interpretation requires baseline values, sampling time, exercise type, soreness, and functional recovery because of large inter-individual variability and delayed kinetics [30].
**Complementary muscle-stress marker**	Myoglobin	Muscle oxygen-binding protein; tissue disruption; early muscle-stress response	May rise after prolonged or muscle-damaging exercise and can reflect acute muscle stress or tissue disruption.	Not a metabolite; included as a complementary tissue-stress marker. Provides information distinct from creatine kinase because of different release and clearance kinetics, but requires temporal sampling and renal-function context [31].
**Core metabolite: glycolytic-oxidative transition**	Pyruvate	Glycolysis; pyruvate-lactate redox balance; entry into oxidative metabolism	May reflect carbohydrate flux and the balance between glycolytic and oxidative metabolism during or after exercise.	Directly measurable but pre-analytically labile. Most informative when interpreted with lactate, glucose, respiratory exchange, exercise intensity, sampling time, and rapid sample handling [13,14,15,16].
**Mechanistic mitochondrial intermediate**	Acetyl-CoA	Link between glycolysis, beta-oxidation, ketone metabolism, and the TCA cycle	Reflects convergence of carbohydrate, lipid, and ketone-derived carbon into mitochondrial metabolism.	Biologically central but difficult to quantify directly in most routine biofluid workflows. Interpretation is usually mechanistic or inferred from related metabolites such as citrate, acylcarnitines, ketone bodies, and TCA-cycle intermediates [19,20,21].
**Core metabolite: TCA-cycle metabolism**	Citrate	TCA cycle; mitochondrial oxidative metabolism; lipid biosynthesis	May reflect changes in mitochondrial flux, substrate oxidation, or recovery-related energy metabolism.	Measurable in targeted metabolomics workflows, but should be interpreted with other TCA-cycle intermediates rather than alone. Requires attention to matrix, sampling time, analytical platform, and pathway consistency [19,20,21].
**Core metabolite: ketone body metabolism**	Beta-hydroxybutyrate (βHB)	Ketogenesis; fatty acid oxidation; carbohydrate availability; metabolic flexibility	May increase during prolonged exercise, fasting, low-carbohydrate availability, ketogenic adaptation, or recovery under altered substrate conditions.	Directly measurable and useful for interpreting lipid-derived fuel use and carbohydrate availability, but not specific to exercise adaptation. Requires dietary control and integration with glucose, free fatty acids, acylcarnitines, and exercise duration [17,18].
**Metabolite class: fatty acid oxidation/mitochondrial stress**	Acetylcarnitine and acylcarnitines	Mitochondrial beta-oxidation; carnitine shuttle; substrate pressure	May reflect mitochondrial substrate load, fatty acid oxidation, incomplete oxidation, or recovery-related substrate redistribution.	Directly measurable, especially by LC-MS-based targeted panels. Interpretation depends on exercise duration, carbohydrate availability, training status, and acylcarnitine chain length; panel-based validation is required [20,21].
**Metabolite class: TCA-cycle/amino acid interface**	Alpha-ketoglutarate, fumarate, oxaloacetate and related intermediates	TCA cycle; anaplerosis; amino acid metabolism; mitochondrial flux	Changes may reflect altered oxidative metabolism, amino acid entry into the TCA cycle, recovery kinetics, or metabolic remodeling.	Some intermediates are measurable by targeted workflows, but labile species such as oxaloacetate require careful pre-analytical handling, rapid processing, matrix-specific validation, and pathway-consistent interpretation [19,20,21].

**Note:** Table 2 is intentionally defined as a broader framework for metabolites and complementary physiological markers. Lactate, glucose, pyruvate, beta-hydroxybutyrate, amino acids, acylcarnitines, citrate, and selected TCA-cycle intermediates are metabolites measurable by appropriate metabolomics workflows, whereas cortisol, IL-6, creatine kinase, and myoglobin are complementary endocrine, inflammatory, and muscle-stress markers. Acetyl-CoA is included as a mechanistically central mitochondrial intermediate but is difficult to quantify directly in most routine biofluid workflows; its interpretation is often inferential. Labile intermediates such as oxaloacetate require careful pre-analytical handling and targeted validation. Each row includes representative supporting references, including glycolytic and carbohydrate-related metabolism [13,14,15,16], ketone-related metabolism [17,18], acylcarnitines and TCA-cycle-related metabolism [19,20,21], amino acids and BCAAs [20,22], endocrine and inflammatory markers [23,24,25], tryptophan-kynurenine metabolism [26,27,28,29], and muscle-stress markers [30,31].

**Table 3:** Evidence-directness and sport-applicability framework for interpreting exercise and sports metabolomics studies.

Evidence Level	Assignment Criteria and Example Study Types	Appropriate Inference	Main Limitations	Validation Requirements
**Level 1: athlete-centered human evidence**	Trained or competitive athletes; sport-relevant exercise or training exposure; defined sampling windows; metabolomics linked to training load, recovery, substrate use, fatigue, adaptation, or performance-related outcomes.	Most appropriate for sport-specific pathway interpretation, candidate biomarker-panel prioritization, and applied hypotheses when analytical quality and repeated-measures design are adequate.	Often limited by small cohorts, sport specificity, sex imbalance, nutrition and recovery heterogeneity, and difficulty separating acute load from chronic adaptation. Circulating metabolites still integrate multiple tissue sources.	Independent athlete cohorts; standardized workload and recovery windows; sport-, sex-, and training-phase-specific analyses; cross-matrix or cross-platform confirmation; linkage to predefined physiological or performance outcomes [1,5,34,35,36,37,38].
**Level 2: controlled healthy-exercise evidence**	Healthy non-athlete or recreationally active participants; controlled acute or training interventions; standardized exercise intensity, duration, nutrition, and sampling; laboratory-based physiological measures.	Supports general physiological interpretation of exercise-responsive pathways and helps define candidate metabolites for testing in athlete populations.	Limited direct generalizability to trained or elite athletes. Responses may differ by training status, sport discipline, sex, environmental exposure, and recovery kinetics.	Replication in trained populations; repeated-measures designs; harmonized metadata; validation across matrices and platforms; assessment against functional outcomes relevant to sport [5,20,34,35,36,37,38].
**Level 3: indirect mechanistic evidence**	Disease, aging, preclinical, tissue-specific, cell, exercise-mimetic, microbiome, guthost, technology-development, or analytical-method studies.	Useful for mechanistic plausibility, pathway prioritization, annotation support, and hypothesis generation.	Should not be used as direct evidence for athlete monitoring, performance optimization, or recovery decisions. Tissue, species, disease-state, and model differences may change metabolic meaning.	Translation requires confirmation in controlled human exercise studies and athlete-centered cohorts; standardized sampling; targeted validation; demonstration that circulating signals reflect relevant physiology rather than nonspecific tissue or matrix effects [57,58,59,87,88,89].

**Note:** Table 3 is supported by the evidence-boundary literature cited in Section 3.2, including athlete-centered and controlled human exercise metabolomics [1,5,20,34,35,36,37,38], indirect mechanistic and multi-omics evidence [57,58,59,87,88,89], and validation standards for annotation, quality control, repeated-measures interpretation, and outcome linkage [32,33,38].

**Table 4:** Key research gaps and validation priorities in exercise and sports metabolomics.

Research Gap	Current Limitation	Evidence Maturity/ Readiness Level	Methodological and Standardization Needs	Validation Priority and Supporting References
**Inconsistent sampling and analytical protocols**	Differences in sample collection, biofluid handling, storage, extraction, analytical platforms, normalization, and batch correction limit cross-study comparability.	Analytical-validation barrier	Harmonize protocols for blood, plasma, serum, urine, saliva, sweat, breath, and tissue samples; define fasting status, exercise timing, collection windows, storage conditions, extraction methods, pooled QC samples, internal standards, and batch-effect correction.	Improve reproducibility across studies, laboratories, matrices, and platforms, and reduce misinterpretation of technical or pre-analytical variation as exercise-induced biology [32,33,38].
**Incomplete metabolite annotation and identification evidence**	Many studies report exercise-responsive features without clearly distinguishing putative annotation from confirmed metabolite identity, limiting pathway interpretation and biomarker-panel development.	Discovery-to-analytical-validation barrier	Report annotation levels transparently; reserve identification for metabolites supported by MS/MS spectra, retention-time matching, authentic standards, isotope-labeled standards where appropriate, collision cross-section values where applicable, and cross-platform confirmation.	Strengthen the transition from untargeted feature discovery to putatively annotated signals, confidently identified metabolites, pathway-linked signatures, and targeted validation panels [32,33].
**Limited longitudinal and sport-specific validation**	Many studies rely on small cohorts, short follow-up periods, isolated post-exercise samples, or heterogeneous athlete groups.	Longitudinal-validation barrier	Use repeated-measures designs across baseline, acute loading, recovery, tapering, competition, travel, environmental exposure, and return-to-training phases; include sex-, sport-, training-status-, and matrix-specific analyses.	Establish within-individual variability, context-specific reference ranges, recovery trajectories, and reproducible metabolite panels across sport types and training phases [20,34,35,36,37,100].
**Unresolved effects of dietary strategies and energy availability**	Carbohydrate periodization, ketogenic or low-carbohydrate diets, intermittent fasting, supplementation, and low energy availability can reshape metabolite profiles, but dietary intake is often insufficiently controlled.	Context-control and longitudinal-validation barrier	Combine metabolomics with validated dietary records, standardized pre-sampling nutrition, assessment of energy availability, and objective markers of substrate use, glycogen availability, ketone metabolism, and energy balance.	Distinguish diet-induced metabolic changes from training adaptation, incomplete recovery, or altered metabolic flexibility [101,102,103].
**Under-characterized environmental and logistical stressors**	Heat, altitude, humidity, travel, jet lag, sleep disruption, and competition stress can influence substrate metabolism, hydration, oxidative stress, inflammation, and recovery.	Context-control and ecological-validation barrier	Record environmental exposure, sleep, travel, hydration, temperature, altitude, humidity, and competition context; design ecologically valid studies with standardized sampling before, during, and after exposure.	Identify metabolite patterns that distinguish adaptive acclimatization from excessive physiological strain, impaired recovery, or increased illness/injury vulnerability [104,105].
**Limited characterization of chronic training adaptation**	Acute exercise responses are better described than long-term adaptations such as mitochondrial remodeling, substrate flexibility, lipid utilization, inflammatory resolution, and sustained metabolic plasticity.	Longitudinal-validation barrier	Conduct longitudinal studies across training cycles; integrate metabolomics with training load, recovery kinetics, performance testing, health status, athlete-reported outcomes, and repeated functional assessments.	Clarify which metabolite trajectories reflect durable adaptation, maladaptation, or recovery status rather than transient acute responses [5,11,106].
**Insufficient integration across omics and physiological data**	Multi-omics studies remain limited by inconsistent metadata, small sample sizes, heterogeneous data structures, and weak linkage between circulating metabolites and tissue-level biology.	Mechanistic-integration and model-validation barrier	Harmonize metadata across metabolomics, lipidomics, proteomics, transcriptomics, epigenomics, microbiome data, and physiological measures; apply interpretable data-integration frameworks with external validation.	Link circulating metabolite patterns to tissue-specific pathways, inter-organ crosstalk, and mechanistic networks of exercise adaptation [57,58,59].
**Uneven validation of wearable, near-real-time, and AI-assisted approaches**	Wearable sensors and computational models are promising but limited by sensor drift, matrix-specific bias, small datasets, overfitting, incomplete metadata, and insufficient external validation.	Implementation-readiness barrier	Calibrate wearable signals against laboratory assays; validate analyte-specific performance across exercise intensity, hydration, environment, and sampling sites; report model development, feature selection, missing-data handling, confounder control, and external validation.	Position wearable and AI-assisted methods as validation-dependent tools for context enrichment and hypothesis generation, rather than standalone metabolomic biomarkers or autonomous decision systems [47,48,49,50,51,52,53,54,55,56,90,91,92,93,94,95,96,97,98,99].

**Note:** Table 4 prioritizes research gaps according to evidence maturity rather than treating them as equivalent issues. Discovery-stage gaps mainly require clearer reporting and hypothesis generation. Analytical-validation gaps require transparent annotation, stronger identification evidence, quality-control assessment, and cross-platform reproducibility. Longitudinal-validation gaps require repeated-measures designs, within-subject baselines, appropriate longitudinal modeling, and outcome linkage. Implementation-readiness gaps require external validation, feasibility testing, and evidence of added value beyond established monitoring measures [5,20,34,35,36,37,38,96,97,98,99,100,101,102,103,104,105,106,107,108,109,110,111].

**Table 5:** Roadmap for evidence-ready exercise and sports metabolomics.

Validation Stage	Purpose	Required Analytical Evidence	Biological/Statistical/Modeling Evidence	Expected Output	Readiness Level
**Stage 1: discovery**	Identify exercise-responsive features and pathway hypotheses.	Transparent preprocessing; missingness and batch-effect assessment; pooled QC performance; annotation confidence reported.	Defined exercise protocol, matrix, sampling time, diet and hydration context, and exploratory pathway interpretation.	Hypothesis-generating feature list or putatively annotated pathway signal, not a biomarker panel.	Discovery level.
**Stage 2: analytical validation and independent replication**	Confirm candidate metabolite identity and measurement robustness.	MS/MS spectra, retention-time matching, authentic standards, internal standards where appropriate, targeted or semi-targeted quantification, analytical precision, QC performance, and explicit distinction between annotation confidence and confirmed identification.	Replication across independent cohorts, matrices, laboratories, or analytical platforms; predefined confounder control.	Confidently identified and quantitatively reproducible candidate metabolite or pathway-linked candidate panel.	Validation-ready candidate, not yet application-ready.
**Stage 3: longitudinal validation**	Determine whether candidate signals track within-individual physiology across training and recovery.	Reproducible assays across batches and time; cross-platform confirmation where feasible; matrix-specific stability.	Repeated-measures reliability; within-subject baselines; mixed-effects or longitudinal modeling; standardized workloads and recovery windows; linkage to predefined physiological or performance outcomes.	Context-specific trajectory, reference range, or longitudinally validated candidate panel.	Longitudinally validated panel requiring external validation before applied use.
**Stage 4: implementation testing**	Assess practical utility and added value in applied sport or exercise settings.	Feasible sampling and turnaround; robust QC in real-world workflows; targeted assay validation under field constraints.	External validation; model calibration and generalizability; sport-, sex-, matrix-, and training-phase-specific thresholds; added value beyond training load, HRV, perceived exertion, sleep, diet, and performance testing.	Application-ready biomarker panel or validated interpretation framework only after implementation testing.	Implementation-ready only after demonstrated added value beyond established monitoring measures.

**Note:** Table 5 presents a staged evidence-readiness framework for exercise and sports metabolomics. Discovery-stage features are hypothesis-generating, whereas application-ready use requires appropriate identification, analytical and longitudinal reproducibility, external validation, outcome linkage, practical feasibility, and added value beyond established monitoring measures. Requirements should be adapted to the biological question, sport context, sample matrix, and intended application.

## Data Availability

No new data were created or analyzed in this study. Data sharing is not applicable to this article.
